# Patterns and Characteristics of Midface Fractures in North-Eastern Romania

**DOI:** 10.3390/medicina59030510

**Published:** 2023-03-06

**Authors:** Andrei-Mihail Roșu, Florentina Severin, Oana Cristina Roșu, Bogdan Mihail Cobzeanu, Stefan Gherasimescu, Florin Petrică Sava, Dragoș Octavian Palade, Cristian Ilie Drochioi, Victor Vlad Costan, Mihail Dan Cobzeanu

**Affiliations:** 1 Surgical Department, Faculty of Medicine University of Medicine and Pharmacy “Grigore T. Popa”, 700115 Iași, Romania; 2 Emergency Clinical County Hospital, 730006 Vaslui, Romania

**Keywords:** midface fractures, interpersonal violence, trauma, maxillofacial fracture

## Abstract

Midface fractures are common injuries that are the result of interpersonal violence, traffic accidents, falls, work-related accidents, sports-related accidents, or animal aggression. In the northeastern part of Romania, these injuries are a significant health concern that, if left untreated, may lead to functional and esthetic sequelae. *Background and Objectives*: This study aims to update the statistical data available to help promote a different lifestyle, with awareness campaigns to prevent aggression, accidents, and domestic violence. *Materials and Methods*: This research was conducted over five years and included 651 patients of both sexes, with ages between 3 and 95 years, that addressed our center for midface fracture treatment. *Results*: The authors of this study found that men are more predisposed to fractures of the middle third of the face, with anterior laterofacial fractures being the most common type of fracture. Interpersonal violence was the most incriminated etiology for all midface fractures. *Conclusions*: The present study regarding midfacial fractures shows similar results compared to the medical literature. These findings could help promote a different lifestyle, with awareness campaigns to prevent aggression, accidents, and domestic violence.

## 1. Introduction

Traumatic pathology is the main cause of mortality in adults under 40 years of age, and a significant part of trauma cases are in the maxillofacial area [1,2].

Of all the injuries that can result after trauma to the cephalic extremity, midface fractures represent an important medical and social problem due to the frequency, complexity and, socio-economic impact they involve. They can have multiple consequences, both aesthetic and functional. In addition to facial deformity, they can cause a malocclusion, difficulty mobilizing the mandible with masticatory problems, diplopia, epiphora, nasal obstruction, respiratory disorders, but also sensory disorders or paresthesia [2,3]. 

Midface fractures are a common type of injury that can occur due to various causes, such as falls, interpersonal violence, car accidents, and sports injuries. These fractures can affect the nose, cheekbones, and maxillary bone and can cause significant physical and emotional distress for the affected individuals, including facial deformities, functional impairment, and long-term scarring. 

In the northeastern part of Romania, midface fractures are a significant health concern, with a high incidence rate among the population. This is likely due to a combination of factors, including the prevalence of high-risk activities and certain factors in the region, such as a high rate of alcohol consumption.

Understanding the epidemiology of midface fractures in the northeastern part of Romania is essential for developing effective prevention and treatment strategies to address this health issue.

In the last years, there has been an increasing interest in understanding the prevalence and patterns of midface fractures in different populations. Therefore, it was considered necessary to carry out a retrospective descriptive statistical study that aims to update the epidemiological characteristics of midface trauma between the years 2015 and 2020, also including the general lock-down period during the COVID-19 pandemic in the northeastern area of Romania.

Midface fractures represent a significant medical and social problem due to their frequency, complexity, and socio-economic impact. It was stated that severe midface fractures protect the brain and torso from major traumatic injuries by dissipating the energy of the impact. A study conducted in the United States over the period 1989–2013, including 20,971 patients with trauma, concluded that severe midface fractures were associated with lower rates of hemorrhagic brain injuries and lower rates of thoracic and abdominal post-traumatic complications [4].

In most cases, a multidisciplinary approach is required, as well as modern diagnostic methods and innovative surgical techniques. In the era of technological medicine and permanent advances, the three-dimensional reconstruction of affected structures based on advanced medical imaging is being discussed, with the aim of more thorough and efficient preparation of operative steps [5].

In the present study, the authors aimed to investigate the prevalence of midface fractures in the northeastern part of Romania. This region has a diverse population with a mix of urban and rural areas, and previous studies have shown that the incidence of midface fractures can vary significantly between different regions. Therefore, this study aims to contribute to a better understanding of the epidemiology of these injuries and to aid the development of preventive measures and treatment strategies.

## 2. Materials and Methods

Within the Emergency Clinical Hospital “Sf. Spiridon” Iași, a retrospective study, aims to establish the epidemiological data from 2015 to 2020 related to midface fractures. Thus, the data on the background, environment, sex, and age of the patients who were treated in the hospital for fractures of the middle third of the face was collected, as well as the type of fracture, the etiology, the need for surgical treatment and the necessary hospitalization period.

Laterofacial fractures interest the zygoma and the zygomatic arch. Centrofacial fractures affect the nasal skeleton and the upper frontomaxillary processes. Oclusofacial fractures are also known as LeFort fractures. LeFort type I fracture affects the anterior maxilla, lateral nasal wall, and pterygoid plates. The LeFort type II fracture line passes through the nasal bones, causing fractures along the nasal bridge, frontal maxilla, lacrimal bones, orbital floor and inferior rim near the inferior orbital foramen, through the anterior wall of the maxillary sinus, and through the pterygoid plates. Lefort type III fractures determine the separation of the midface from the base of the skull, and the fracture line affects the nasal bridge, the medial orbital wall, the orbital floor, passes along the lateral orbital wall, through the zygomatic arch, ethmoid bone, and pterygoid processes.

A number of 651 subjects aged between 3 and 95 were included, patients of both sexes who suffered a midfacial trauma.

This study was carried out with the approval of the “Sf. Spiridon” Iași Emergency Clinical Hospital ethics committee, as well as of the “Grigore T. Popa” University of Medicine and Pharmacy Iași, in compliance with the European General Data Protection Regulation (GDPR) convention and the legislation in force on the protection of personal data.

The data was analyzed statistically using IBM SPSS Statistics 26 (IBM Corp. Released 2019, IBM SPSS Statistics for Windows, Version 26.0. Armonk, NY, USA: IBM Corp.) and Microsoft Excel 2023 (Microsoft Corporation. (2023), Microsoft Excel for Mac, Version 16.70, Redmond, Washington, United States. Retrieved from https://office.microsoft.com/excel, accessed on 21 January 2023) using descriptive statistic (average values, maximum values, 25th and 75th percentiles respectively), the ANOVA test, chi-square test and the study of the correlation between different phenomena was carried out using the correlation coefficient r (Pearson).

## 3. Results

Out of the total number of patients included in the study, 87 were females, representing 13.36%, and 564, respectively 86.63%, were males; with a distribution of 6.48 to 1 in favor of the male sex with the mean age for female participants being 46.83 (min—5 years, max—95 years) and 40,49 for the male group (min—3 years, max—89 years) 

**Figure 1:**
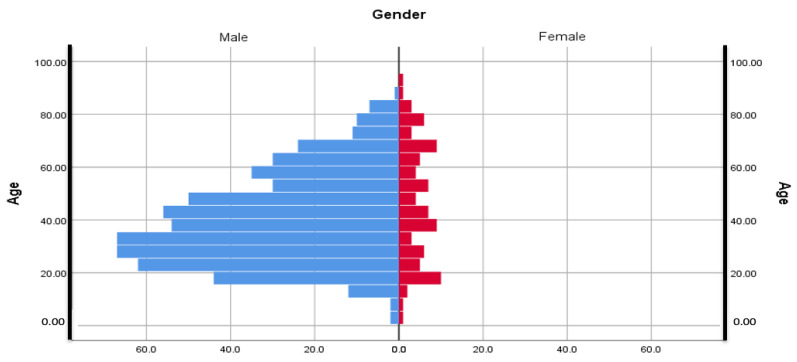
Distribution of the studied lot according to gender and age.

Additionally, the age groups 21–30 years (140 patients), 31–40 years (133 patients), and 41–50 years (117 patients) prevailed in the case of midface traumas. Additionally, 45.01% (mean age 40.97; CI 38.82–43.11; Min 5; Max 95) of subjects were from an urban environment, with 38.25% being male and 6.76% female, while 54.99% (age mean 41.33; CI 39.52–43.13; Min 3; Max 89) were from the rural area, of which 48.39% males and 6.51% females (Figure 1). From the studied lot, a total of 462 (70.81%) patients admitted to our service required surgical treatment, while 189 (29.19%) were treated conservatively (Table 1).

**Figure 2:**
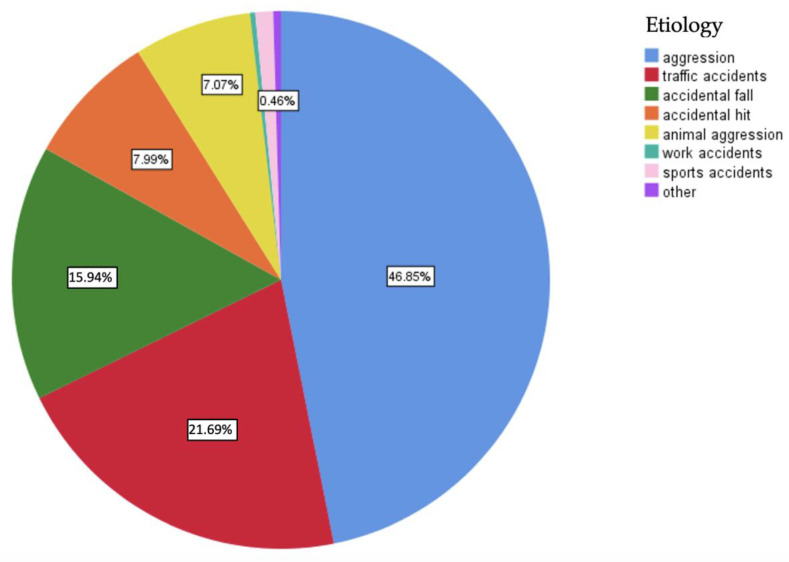
Chart reflecting the etiology of midface trauma in the studied lot.

**Figure 3:**
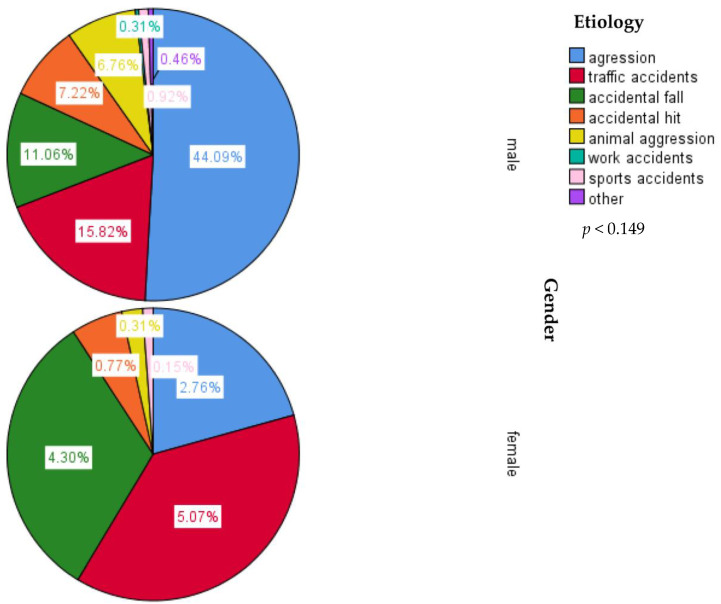
Etiology for midface trauma between gender.

Regarding the etiology of midface trauma, the first place was occupied by interpersonal violence, representing 46.85% of cases, followed by traffic accidents. In the last places, we found work and sports-related accidents (Figure 2). When the lot was divided according to gender, the predominance of interpersonal violence was maintained in the case of male participants. In contrast, for most female participants, midface trauma was caused by traffic accidents (Figure 3).

When comparing the number of hospital attendance by gender for the COVID-19 pandemic lock-down period with the same period from the previous year, the authors found no significant differences between female and male patients that were admitted to the hospital (*p* = 0.886) with a total number of patients of 16 admitted between march and may 2019 and 14 for the same period in 2020.

**Figure 4:**
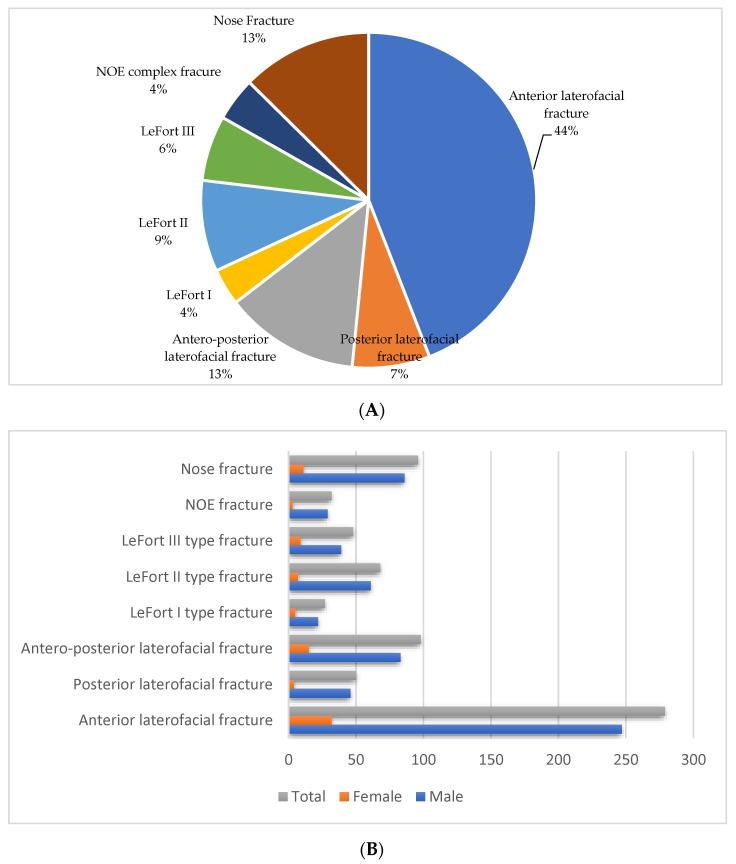
(**A**)Types of midface fractures represented as a percentage, (**B**) Types of midface fractures according to gender.

**Figure 5:**
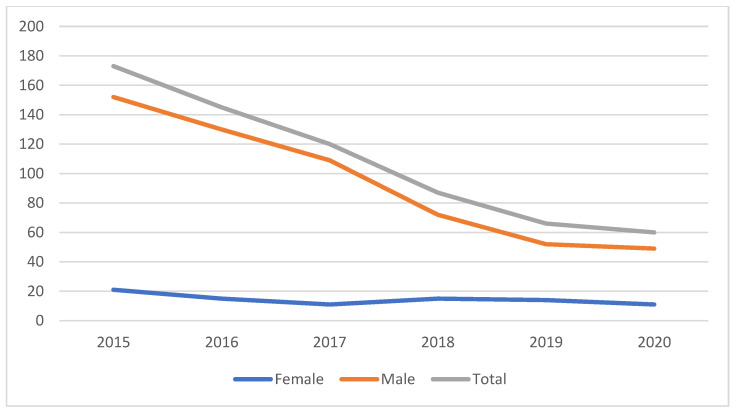
The number of cases of midfacial trauma over the years of the studied period.

As for the different types of midface fractures, our study found that the most frequent, representing 44% of the total cases, were anterior laterofacial fractures, followed by anteroposterior laterofacial fractures and nose fractures, each representing 13%. On the other hand, the least encountered types of midface fractures were NOE (naso-orbio-ethmoidal) complex and LeFort type I fractures (Figure 4A,B). In addition, this study found that hospital admission for midface fractures had a continuous drop over the studied period, although the number of female patients was similar for each year in the documented period (Figure 5).

## 4. Discussion

Our study found that most of the midface trauma cases were anterior laterofacial fractures representing 44%, followed by anteroposterior laterofacial fractures and nose fractures, each representing 13%. LeFort type II and LeFort type III fractures expressed 9% and respectively 6% of the total studied lot, with LeFort type I and NOE complex fractures each representing 4% of the cases. Men were especially at risk, surpassing the female patients, with a ratio of 6.48 to 1, with adults between 21 and 50 years old comprising more than 80% of the total patients included in this study. 

The values of male to female ratio differ greatly in the specialized literature, depending on the area where the studies were conducted. Thus, a lower value of only 1.8:1 was recorded in Italy in 2017 in a study that followed trauma in the maxillo-facial region over a period of 15 years and included 1720 patients in the research [6]. Another study carried out in Europe, this time in Austria in 2003, reported a ratio of 2.1:1 (male: female) [2]; in Amsterdam, in 2013, the stated ratio was 2.6:1 [1], and in China, the male: female ratio was 3.5:1 [7]. A ratio similar to that obtained in our study (6.4:1) was reported by a survey conducted in the Arab countries around the Persian Gulf in 2021, which included more than 19,000 patients [8]. Higher values with a ratio of 8:1 were reported by India [5], but also in Africa, where this ratio reaches 12:1 in certain areas [9]. These large differences between the number of men and women with facial fractures could be explained by socio-economic, cultural, and educational factors. 

A study realized in 2015 comparing data from multiple centers enrolled in the European Maxillofacial Trauma project about the demographics, cause, and characteristics of maxillofacial trauma showed a male-to-female ratio of 3.6:1 overall. Still, the ratio varied from center to center, with the highest ratio (9,4:1) present in Kyiv, Ukraine, and the lowest in Amsterdam, The Netherlands (2.2:1). Additionally, the mean age varied from 29.9 years in Dundee, Scotland, UK to 43.9 in Ljubljana, Slovenia. Interpersonal violence (39%) was the most incriminated etiology, followed by falls (31%), traffic accidents (11%), sport-related injuries (11%), and work-related injuries (3%) [10].

Regarding the etiology of midface traumas, for the entire studied group, the first place was occupied by interpersonal violence with 46.85% of cases, followed by traffic accidents at 21.69%, accidental falls at 15.94%, accidental hits at 7.99%, and aggression caused by animals with a percentage of 7.07%. In the last places, we found accidents at work and those resulting from sports activities. After dividing the group according to gender, we noticed that, for male participants, the situation reflected the global trend, with aggression being by far the most frequent etiology, followed by traffic accidents, falls, and accidental hits, while for female patients, the most cases of midface trauma were caused by traffic accidents, followed by accidental falls and, in 3rd place, by assault. Regarding the etiology of these fractures in the general population and, subsequently, by gender, numerous studies have been conducted worldwide at different time periods. We can thus try to outline an etiological hierarchy of midfacial fractures, although the results of the studies are extremely varied from one geographic region to another.

In European countries, interpersonal violence is also the most incriminated etiology, but with a lower percentage than that objectified by our study (39% vs. 46.85%), followed by falls (31%), traffic accidents (11%), sports-related injuries (11%) and work accidents (3%) [10]. Other countries reported different results, as follows: in the Netherlands (2013), the most frequent etiology was represented by traffic accidents, regardless of the patient’s gender, followed by interpersonal violence for men, respectively, by falls in the case of women. For those who consumed alcohol, aggression was most frequently incriminated [1]. On the other hand, in Italy in 2017, in a study carried out over a period of 15 years on 1720 patients, the hierarchy of etiologies looked like this: in first place were road accidents (57.1%), followed by interpersonal violence (21.7%), falls (14.2%), work accidents (3.5%), respectively sports accidents (3.3%) [6]. Another study from Italy conducted in 2013 also mentions road accidents as the main etiology of facial fractures [11]. We find that there are differences depending on the specifics of the area; thus, in Austria in 2003, a study carried out on a group of 9543 patients described a completely different order of etiologies: initially, daily activities were incriminated (38%)—including falls, followed by sports accidents (31%), then interpersonal violence and traffic accidents, each with 12%, 5% work accidents, and 2% other causes. 19% of the patients included in the study were foreigners, with the Austrian Alps being a highly frequented area for winter sports [2]. In Croatia, authors reported falls as the main etiology, followed by aggression for men and traffic accidents for women. [12]. The same study also describes a difference regarding age, with interpersonal violence more frequent among young people, with falls being the prerogative of adults over 50.

A study that analyzed the clinical patterns and characteristics of midfacial fractures over a period of 10 years, this time in the western Romanian population, found that the most common types of fracture were laterofacial (50%) and nasal bone fractures (22.93%), with LeFort type fractures being the least encountered, representing 0.83% for LeFort type I, 1.03% for LeFort Type II and 0.83 for LeFort type III from a total of 397 patients [13]. Another study from the same part of the country found that midface fractures affect most commonly the male sex and patients from the urban area (54.35%) aged between 20 and 29 years old, with the most incriminated etiology being assault, followed by fall trauma and road traffic accidents [14]. The fact that the second most incriminated etiology from the north-eastern part of the country is road traffic accidents, and for the western part is accidental falls could be explained by a more developed transport infrastructure in the western part of the country. 

Regarding the background, the current study mentions that 45.01% of the subjects are from urban areas, while 54.99% are from rural areas. Another interesting observation would be the similar percentage of women from both backgrounds (6.76% in the urban environment versus 6.51% in the rural environment), thus not supporting the statement that domestic violence is more frequent in the rural environment. On the other hand, there is a greater number of men coming from the rural environment, 48.39% of the whole lot, compared to 38.25% from the urban environment. This can be explained by the level of education, the more frequent consumption of alcoholic beverages, but also their involvement in raising animals or other activities within the household. 

Our study’s results showed a continuous drop of the hospital attendance rate for midface fractures over the studied period. This can be attributed to a better implementation of public safety rules, better management of alcohol consumption and stricter traffic regulations, the implementation of a better diagnostic protocol utilizing cone beam computer tomography instead of conventional radiographic studies, and the cases of maxillofacial trauma being directed to other healthcare centers in the region. This phenomenon may also be explained by the lawmaker’s concern for citizens’ safety, whcih led to tougher sentences for acts of aggression (including interpersonal violence and road traffic accidents) directed to members of the family or for those committed by inebriated authors [15].

The authors compared the data regarding hospital attendance for midfacial trauma during the COVID-19 general lockdown with the same period of the previous year, but the results showed no significant difference. In contrast, another recently published study concerning the effect of the COVID-19 pandemic on midface fractures found a decrease in hospital admissions for facial trauma, approximately seven times lower, comparing the number of patients from March to April 2019 with the same period in 2020 [16]. 

Interpersonal violence was the most incriminated cause of midface trauma, followed by traffic accidents and accidental falls. It must be noted that men were more affected by violence, while the leading cause for women was traffic accidents. Other studies in the literature show a different predominance in the etiology of facial fractures: an Indian study conducted in 2019 on 944 patients with facial trauma, with 19% midface fractures, found that the leading cause was by far road traffic accidents [17]. The same predominance of road traffic accidents is mentioned in another study from Saudi Arabia (2019) [18] and India (2022) [19].

The current study also underlines the predominance of young male adults, with a peak incidence between the 2nd and 3rd decade of life. Most studies report the same conclusions, with young people in their 20s and 30s having an active lifestyle and engaging in many outdoor activities [8]. The average age for female patients was 46.83 years (minimum age—5 years, maximum age—95 years) and 40.49 years for male patients (minimum age—3 years, and maximum age—89 years). In general, most studies on the epidemiology of fractures in the oro-maxillo-facial region describe an older average age for female patients, something also highlighted in a study from Italy from 2017, where the average age for women with facial fractures is 59.5 years [6]. For Europe, a varying average age of the whole lot can be observed, regardless of sex, from 29.9 years in Dundee, Scotland, UK, to 43.9 in Ljubljana, Slovenia [10]. Reviewing data from the literature, we find that the predominance of young adults in the group of patients with facial trauma has not changed over time, being described in Sweden in 1980 [20] and in Scotland (1985) [21]. 

Although children are more prone to craniofacial trauma, the absence of sinus pneumatization, the bone elasticity, the thickness of the periosteum, and the retruded position of the face in relation to the neurocranium offer a greater degree of protection [22]. In our study, only 58 patients were children (maximum age of 18 years), representing 8.91% of the entire lot. A total number of 38 children received surgical treatment, representing 65,51%. A smaller percentage was mentioned in a study conducted in Indianapolis, Indiana, in 2019, on 218 pediatric patients, showing that only a quarter (25.2%) of the hospitalized children needed surgical intervention, the rest receiving conservative treatment [23]. 

Craniofacial trauma is common in all age groups. The cause is closely related to age, sex, and alcohol consumption, and it determines the type and severity of the injury. Hussain states that accidental falls are the primary cause of injuries in elderly patients, while interpersonal violence and traffic accidents are responsible for injuries in patients between 15 and 50 years of age. Physical violence most often involves young adults, and fights usually occur between strangers who have consumed excessive amounts of alcohol. Women are assaulted by people known to them, in most cases, their life partners; pedestrians are prone to skull fractures, vehicle occupants involved in traffic accidents suffer midfacial fractures, and cyclists have mandibular fractures [24].

Our study found that the most common types of fractures affecting the midface were those involving the zygomatic complex, namely laterofacial fractures (75.58%) followed by oclusofacial fractures (LeFort type fractures) (21.81%) and centrofacial fractures (19.66%). Other authors reported that fractures of the zygomatic complex accounted for 62.5% of the total number of midface fractures, followed by LeFort II type fractures (23%), multiple fractures of the midface (10%), LeFort I type fractures (6%), LeFort III fractures (4.5%), and naso-orbito-ethmoid complex fractures (4%) [25].

Fractures of the NOE complex are the result of forces applied to the middle third of the face. Due to the violence of the impact required to cause these fractures, other facial, cranial, or body lesions may be present. Traffic accidents, more often those involving an occupant not wearing a seat belt at the time of impact, are the most common causes of trauma affecting the NOE complex, representing 4% of all adult skull fractures [26,27], the most frequently affected being young male adults. This corresponds with the results of our study.

Midface fractures’ treatment follows similar principles to the treatment of other systemic fractures but presents a series of particularities due to the complexity of the facial anatomy [28], with authors stating that the restoration of the facial vertical buttresses restores the load-bearing structure of the midface, while the rehabilitation of the horizontal buttresses recovers the aesthetic aspect. 

The authors of this study found that 461 (70.81%) required and received surgical treatment consisting of open reduction and internal fixation with titanium miniplates and/or mesh for selected cases of laterofacial or nose fractures, while 189 (29.19%) patients admitted in our center were treated conservatively. Our results are similar to other studies. Manodph et al. found that 73.56% of the entire lot (3611 patients) required surgery [29], while Wouter et al. reported similar percentages in a tertiary trauma center from the Netherlands where, over a period of 5 years, 293 (74%) patients received surgical treatment for midface fractures [30]. A higher percentage of 85.4% was mentioned in a study from Nepal in 2021 [31] and in Berlin, Germany, where 89.5% of patients with midface fractures were treated surgically in 2019 [32]

## 5. Conclusions

The present study regarding midfacial fractures shows similar results compared to the medical literature, with certain particularities: a male predominance with more than 80% of the current lot, a high incidence of interpersonal violence and traffic accidents, as well as high frequency of anterior laterofacial fractures, compared to other types. In addition, we observed a continuous descending trend in the total number of hospitalizations. 

Most of the patients received surgical treatment. 

Given the increasing esthetic and functional demands, a continuous update of available resources is necessary for a better outcome for midface fracture cases.

Reviewing the literature shows an extremely high variability of etiological agents, influenced by numerous factors such as socio-economic status, cultural background, life habits, or level of education. These findings could help promote a different lifestyle, with awareness campaigns to prevent aggression, accidents, and domestic violence. Further studies are needed for dynamic observation of changes in the epidemiology of midface fractures, to establish the effectiveness of preventive strategies, and also to note the impact of lifestyle on facial traumatic pathology.

## Figures and Tables

**Table 1:** Descriptive indicators depending on the type of treatment.

	Gender	N	%	Mean Age	Std. Dev.	Min.	Max.	*p*
medical treatment	female	32	11.90	47.75	24.20	5	89	0.513
	male	157	88.10	40.19	17.00	3	85	
	TOTAL	189	100.00	41.47	18.53	3	89	
surgical treatment	female	55	16.93	46.30	21.55	13	95	0.102
	male	407	83.07	40.33	17.05	8	89	
	TOTAL	462	100.00	41.04	17.72	8	95	

## Data Availability

Data available on request.

## References

[1] Salentijn E.G., Bergh B.V.D., Forouzanfar T. (2013). A ten-year analysis of midfacial fractures. J. Cranio-Maxillofac. Surg..

[2] Gassner R., Tuli T., Hächl O., Rudisch A., Ulmer H. (2003). Cranio-maxillofacial trauma: A 10 year review of 9543 cases with 21067 injuries. J. Cranio-Maxillofac. Surg..

[3] Yamamoto K., Matsusue Y., Horita S., Murakami K., Sugiura T., Kirita T. (2014). Clinical Analysis of Midfacial Fractures. Mater. Socio Med..

[4] Woriax H.E., Hamill M.E., Gilbert C.M., Reed C.M., Faulks E.R., Love K.M., Lollar D.I., Nussbaum M.S., Collier B.R. (2018). Is the Face an Air Bag for the Brain and Torso? -The Potential Protective Effects of Severe Midface Fractures. Am. Surg..

[5] Satish P., Prasad K., Lalitha R.M., Ranganath K., Sagar P. (2017). Analysis of the Changing Patterns of Midface Fractures Using 3D Computed Tomography: An Observational Study. Craniomaxillofacial Trauma Reconstr..

[6] Bonavolonta P., Orabona G.D., Abbate V., Vaira L.A., Faro C.L., Petrocelli M., Attanasi F., De Riu G., Iaconetta G., Califano L. (2017). The epidemiological analysis of maxillofacial fractures in Italy: The experience of a single tertiary center with 1720 patients. J. Cranio-Maxillofac. Surg..

[7] Zhou H.-H., Liu Q., Yang R.-T., Li Z., Li Z.-B. (2015). Maxillofacial Fractures in Women and Men: A 10-Year Retrospective Study. J. Oral Maxillofac. Surg..

[8] AlQahtani F., Bishawi K., Jaber M., Thomas S. (2020). Maxillofacial trauma in the gulf countries: A systematic review. Eur. J. Trauma Emerg. Surg..

[9] Jaber M.A., AlQahtani F., Bishawi K., Kuriadom S.T. (2021). Patterns of Maxillofacial Injuries in the Middle East and North Africa: A Systematic Review. Int. Dent. J..

[10] Boffano P., Roccia F., Zavattero E., Dediol E., Uglešić V., Kovačič Ž., Vesnaver A., Konstantinovic V., Petrović M., Stephens J. (2015). European Maxillofacial Trauma (EURMAT) project: A multicentre and prospective study. J. Cranio-Maxillofac. Surg..

[11] Roccia F., Boffano P., Bianchi F.A., Ramieri G. (2012). An 11-year review of dental injuries associated with maxillofacial fractures in Turin, Italy. Oral Maxillofac. Surg..

[12] Siber S., Matijević M., Sikora M., Leović D., Mumlek I., Macan D. (2015). Assessment of Oro-Maxillofacial Trauma According to Gender, Age, Cause and Type of the Injury. Acta Stomatol. Croat..

[13] Tent P., Juncar R., Juncar M. (2019). Clinical patterns and characteristics of midfacial fractures in western romanian population: A 10-year retrospective study. Med. Oral Patol. Oral Y Cirugía Bucal.

[14] Tent P.A., Juncar R.I., Lung T., Juncar M. (2018). Midfacial fractures: A retrospective etiological study over a 10-year period in Western Romanian population. Niger. J. Clin. Pract..

[15] Codul Penal al României, 21.06.1968, art. 181, 182, 184; Republicat și Modificat. https://legislatie.just.ro/Public/DetaliiDocument/38070.

[16] Kasem A., Redenski I., Oren D., Zoabi A., Srouji S., Kablan F. (2022). Decline in Maxillofacial Injuries during the Pandemic: The Hidden Face of COVID-19. J. Clin. Med..

[17] Abhinav R.P., Selvarasu K., Maheswari G.U., Taltia A.A. (2019). The patterns and etiology of maxillofacial trauma in South India. Ann. Maxillofac. Surg..

[18] Al-Bokhamseen M., Salma R., Al-Bodbaij M. (2018). Patterns of maxillofacial fractures in Hofuf, Saudi Arabia: A 10-year retrospective case series. Saudi Dent. J..

[19] Menon S., Shivakotee S., Sham M., Kumar V., Archana S. (2022). Midface fracture pattern in a tertiary care hospital—A prospective study. Natl. J. Maxillofac. Surg..

[20] Afzelius L.-E., Rosén C. (1980). Facial fractures: A review of 368 cases. Int. J. Oral Surg..

[21] Ellis E., Moos K.F., El-Attar A. (1985). Ten years of mandibular fractures: An analysis of 2137 cases. Oral Surg. Oral Med. Oral Pathol..

[22] Cole P., Kaufman Y., Hollier L.H. (2009). Managing the Pediatric Facial Fracture. Craniomaxillofacial Trauma Reconstr..

[23] Kao R., Campiti V.J., Rabbani C.C., Ting J.Y., Sim M.W., Shipchandler T.Z. (2019). Pediatric Midface Fractures: Outcomes and Complications of 218 Patients. Laryngoscope Investig. Otolaryngol..

[24] Hussain K.B., Wijetunge D.B.M., Grubnic S.M., Jackson I.T.M. (1994). A Comprehensive Analysis of Craniofacial Trauma. J. Trauma Inj. Infect. Crit. Care.

[25] Bulgaru Iliescu D., Enache A., Scripcaru C., Curcă G. (2021). Tratat de Traumatoligie Medico-Legala.

[26] Kelley P., Crawford M., Higuera S., Hollier L.H. (2005). Two Hundred Ninety-Four Consecutive Facial Fractures in an Urban Trauma Center: Lessons Learned. Plast. Reconstr. Surg..

[27] Cabalag M.S., Wasiak J., Andrew N.E., Tang J., Kirby J.C., Morgan D.J. (2014). Epidemiology and management of maxillofacial fractures in an Australian trauma centre. J. Plast. Reconstr. Aesthetic Surg..

[28] Wusiman P., Maimaitituerxun B., Guli, Saimaiti A., Moming A. (2020). Epidemiology and Pattern of Oral and Maxillofacial Trauma. J. Craniofacial Surg..

[29] Manodh P., Shankar D.P., Pradeep D., Santhosh R., Murugan A. (2016). Incidence and patterns of maxillofacial trauma—A retrospective analysis of 3611 patients—An update. Oral Maxillofac. Surg..

[30] Van Hout W.M., Van Cann E.M., Abbink J.H., Koole R. (2013). An epidemiological study of maxillofacial fractures requiring surgical treatment at a tertiary trauma centre between 2005 and 2010. Br. J. Oral Maxillofac. Surg..

[31] Chaurasia N.K., Upadhyaya C., Dulal S. (2021). Etiology, Pattern, Treatment and Outcome of Maxillofacial Fractures at Dhulikhel Hospital. Kathmandu Univ. Med. J..

[32] Goedecke M., Thiem D.G.E., Schneider D., Frerich B., Kämmerer P.W. (2019). Through the ages-Aetiological changes in maxillofacial trauma. Dent. Traumatol..

